# Effects of glyceryl trinitrate and calcitonin-gene-related peptide on BOLD signal and arterial diameter –methodological studies by fMRI and MRA

**DOI:** 10.1186/1129-2377-14-S1-I4

**Published:** 2013-02-21

**Authors:** MS Asghar, M Ashina

**Affiliations:** 1 Danish Headache Center, Denmark

## Introduction

Over the last decades MRI has proved to be very useful in the field of drug development and discovery. Pharmacological MRI (phMRI) explores the interaction between brain physiology, neuronal activity and drugs[1]. The BOLD-signal is an indirect method to investigate brain activity by way of measuring task-related hemodynamic changes. Pharmacological substances that induce hemodynamic changes can therefore potentially alter the BOLD-signal that in turn falsely can be interpreted as changes in neuronal activity. It is therefore important to characterize possible effects of a pharmacological substance on the BOLD-response per see before that substance can be used in an fMRI experiment. Furthermore MR-angiography is useful in determining the vascular site-of-action of vasoactive substances.

## Methods

Four substances; Acetazolamide, Glyceryl Trinitrate (GTN), CGRP and sumatriptan has been examined using a 3-Tesla MRI scanner in three double-blind placebo-controlled crossover studies in normal volunteers.

## Results

Acetazolamide depresses the BOLD-signal by increasing cerebral blood flow (CBF). GTN had surprisingly no effect on the BOLD-signal even though it is known to increase cerebral blood volume (CBV)[2]. Infusion of CGRP induces immediate headache and dilates the middle meningeal artery (MMA) but contrarily to previous belief does not dilate the middle cerebral artery (MCA)[3]. Nor does CGRP increase brain activity per see[4]. Sumatriptan ameliorates headache, contracts MMA and marginally constricts MCA[3] without altering brain activity[4].

## Conclusion

Acetazolamide depresses the BOLD-signal while GTN does not alter the BOLD-signal. Neither CGRP or sumatriptan has a direct effects on brain activity. Instead it seems that both the migraine provoking peptide CGRP and the anti-migraine drug sumatriptan exert their actions outside of the blood brain barrier. These studies show that phMRI can be a powerful tool in understanding mechanisms and site-of-action of pharmacological compounds. And can have important implication for implementation of fMRI in headache research.
